# Synthetic microbial communities for studying the fate and ecological role of specialized metabolites

**DOI:** 10.1042/EBC20250027

**Published:** 2026-08-19

**Authors:** Ákos T. Kovács

**Affiliations:** Institute of Biology, Leiden University, Leiden, Netherlands

**Keywords:** BGC, bioconversion, chemical ecology, specialized metabolite, SynCom

## Abstract

A wide range of microbially produced specialized metabolites (SMs) with bioactivity affecting micro- and macro-organisms has been described. These bioactive compounds are synthesized by enzyme complexes encoded in biosynthetic gene clusters (BGCs). Historically, the first role of SMs was revealed through antimicrobial activity-based assays. However, diverse bioactivities have since been deciphered that are not associated with inhibition of other organisms. To advance discoveries in chemical ecology, synthetic microbial communities (SynComs), simplified, experimentally tractable systems that recapitulate specific features of natural microbiomes, are increasingly employed. SynComs enable the systematic investigation of SM-mediated induction of BGC expression, the characterization of SM bioconversions, and the elucidation of the mechanism by which SMs influence microbial establishment and persistence within communities. Due to their reduced complexity, SynComs allow the controlled determination of microbial community composition and functional dynamics, as well as the characterization of associated chemical diversity. This review highlights representative publications describing how SynComs are employed to elucidate the roles of SMs in complex microbial interactions and emphasizes the emerging functions observed in SynCom-based chemical ecology studies.

## Introduction

**Figure 1:**
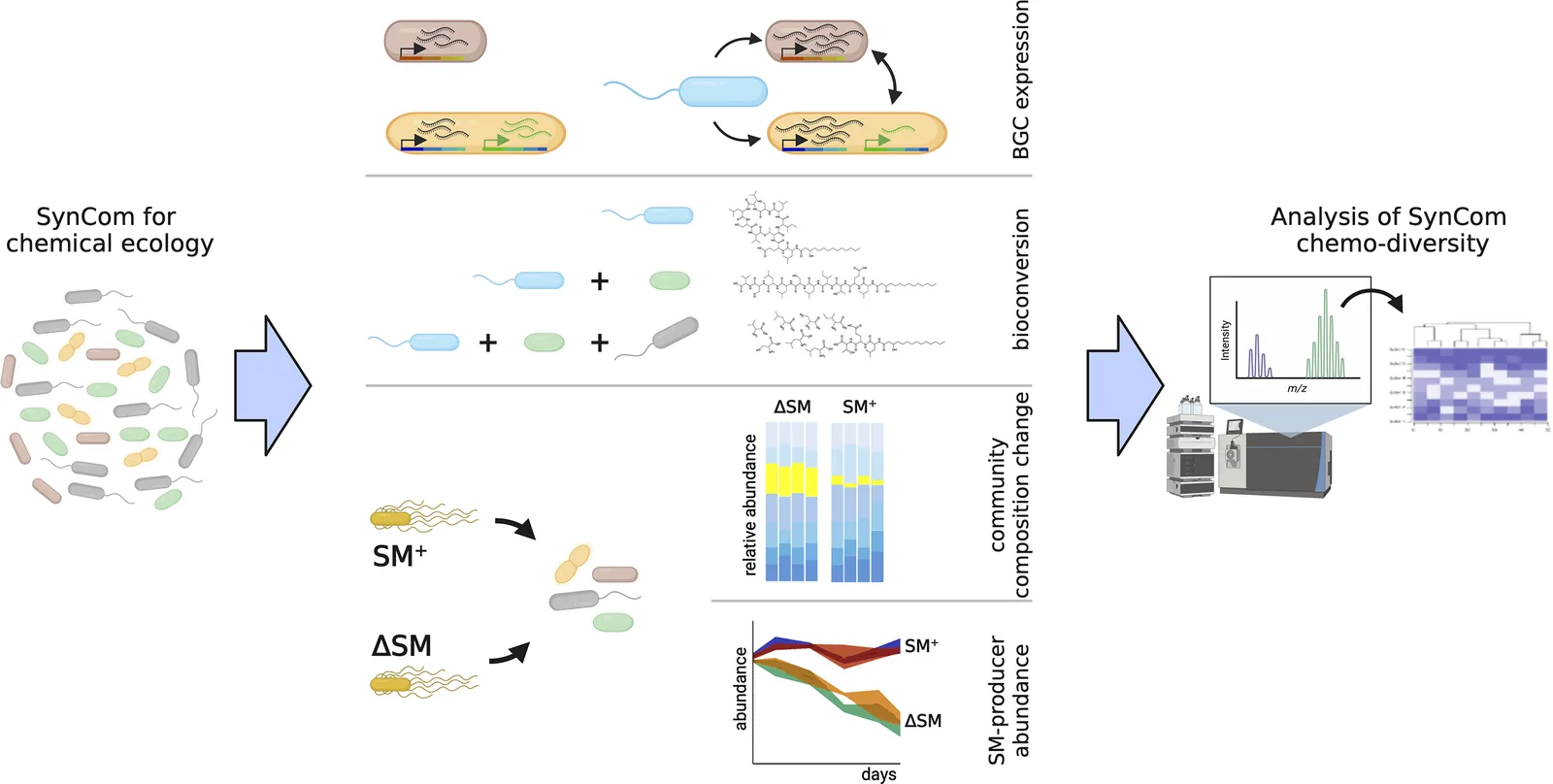
SynComs can be utilized to explore SM-dependent community dynamics Ecological interaction includes (middle panel from top row to bottom) community member-dependent alteration of BGC expression (mRNA symbolized as half ladder), SM bioconversion by community members, influence of SM production on community composition, and importance of SM production on invader abundance in the SynCom. Analysis of SynCom metabolome facilitates the dissection of altered SM dynamics (right panel). The chemical structures used in the bioconversion panel are from reference [38].

Assembly, composition, and dynamics of microbial communities are influenced by interactions among the members. This includes different levels of cooperation and competition. In contrast to direct cell-cell interactions that are dependent on various cell surface structures, primary and specialized metabolites (SMs) enable interactions without close contact between cells in the community. Primary metabolites are frequently implicated in cross-feeding, where different members of the microbial community reciprocate metabolites, which benefit the growth of the community members exchanging the nutrients [1–3]. SMs, organic molecules that are not essential for the normal growth of an organism, have diverse roles. SMs can inhibit the growth of other microorganisms, act as signaling molecules, sustain survival under stress conditions, and influence the differentiation of the producing organism or other organisms, including animal or plant hosts [4–6]. SMs display an enormous diversity both in their structure and mode of action. Diverse approaches are exploited to understand the function of SMs in microbial communities with different levels of complexity, from simple pairwise interactions to the multifaceted influence of an SM-producing organism on the natural microbiome composition and functioning [7–12]. Understanding SM-mediated interactions allows the construction of microbial consortia for various applications, such as protection of plants against pathogens [13,14]. While pairwise interactions on an artificial laboratory medium are excessively simplistic, it is challenging to dissect the mechanistic details of microbiome behavior due to its complexity, especially in a natural environment. Synthetic microbial communities (SynCom) are utilized as tractable systems that recapitulate certain aspects of microbiome composition and characteristics [15–19]. The use of SynCom allows the dissection of the mechanistic background of microbial community performance, higher-level interactions that are not feasible with just pairwise experiments, but SynCom application also permits the reduction of complexity within natural systems. The complications caused by the complex chemical landscape of microbiomes can also be reduced using a SynCom. Simple SynCom-containing systems can be exploited to dissect the influence of these diffusible SMs [20]. This review highlights examples of how SynComs are utilized to investigate the influence of SMs on community assembly and dynamics and to reveal the influence of SynCom composition on the production and amount of SMs, including the induction of SM production and bioconversion by the SynCom members (Figure 1).

## Induction of SM production in microbial communities

SMs can be formed by enzyme complexes or multi-domain enzymes, including non-ribosomal peptide synthetases and polyketide synthases, or they can be ribosomally synthesized and post-translationally modified peptides (RIPPs) [21–25]. The genes encoding SM synthesis are arranged in large biosynthetic gene clusters (BGCs) [26]. The clustering of genes within the BGCs facilitates the identification of genes encoded in the genome of microorganisms [27–30]. While most microbial genomes encode large number of BGCs, only a fraction of these is expressed, limiting the detection of SMs under laboratory conditions [31]. Activation of silent BGC expression facilitates the discovery of novel natural products and drug development [11,31,32]. High-throughput platforms have been developed to identify elicitors of silent BGCs [11], in addition to exploiting our understanding of microbial ecology to awaken the production of cryptic antimicrobials [33]. A plethora of chemical compounds has been identified to induce the expression of BGCs, including primary metabolites and SMs, self-produced and interspecies signaling molecules [6–8,12,31,33–36]. Expression of BGC might be suppressed by self-produced SM; the lipopeptide surfactin of *Bacillus subtilis* represses the production of its RiPP, subtilosin A [37]. Whereas *Pseudomonas capeferrum* induces the production of pyoluteorin in *Pseudomonas protegens*, the secreted pyoluteorin creates lysis of *P. capeferrum* cells, and the released intracellular metabolites subsequently inhibit the production of another SM of *P. protegens*, 2,4-diacetylphloroglucinol [38].

Pairwise interactions among different bacterial isolates are exploited to reveal the induction of SM production [34,39]. For example, *Vibrio coralliilyticus*-produced andrimid, a specialized inhibitor of bacterial fatty acid biosynthesis, increases the expression of several BGCs in *Photobacterium galatheae* possibly through activation of stress response [40]. Similarly, expression of BGCs in *Bacillus velezensis* is induced in the presence of fungi that are dependent on the general stress-related sigma factor, σ^B^ in the bacterium [41]. Indeed, competition sensing has been proposed to play a pivotal role in inducing the expression of defensive traits in microorganisms, including the production of protective biofilm matrix and offensive antimicrobials [42].

SynComs can be utilized to reveal complex interactions among the community members. Chevrette and colleagues used a tractable three-species model community, THOR (the hitchhikers of the rhizosphere), to dissect BGC expression and SM abundance [43]. The THOR community comprises the soybean rhizosphere-derived *Bacillus cereus* and the co-isolated *Pseudomonas koreensis* and *Flavobacterium johnsoniae* strains that interact under field and laboratory conditions and are amenable to genetic analysis [44]. The genomes of these three species contain a total of 100 putative BGC loci encoding the synthesis of characterized and cryptic SMs with antimicrobial and siderophore functions. Metatranscriptomic analysis revealed that in the THOR community, several BGC expressions shift relative to monocultures. Coculture with *P. koreensis* has the most pronounced effect. Many *B. cereus* and *F. johnsoniae* BGCs are differentially expressed in the three-member community, while *P. koreensis* BGC expression is largely unchanged [43]. While expression of the *B. cereus* zwittermicin/kanosamine locus is induced, expression of the bacillibactin BGC is down-regulated in the THOR community, as well as when *B. cereus* co-cultured with *P. koreensis*. Interestingly, expression of the siderophore petrobactin BGC in *B. cereus*-*F. johnsoniae* and *B. cereus–P. koreensis* dual cultures is comparable to that in the monocultures, while it is increased in the three-member community, suggesting a combinatorial community effect that cannot be attributed to a single partner species. Metabolome analysis of the mono-, dual-, and three-member cultures confirmed the increased production of antimicrobial zwittermicin/kanosamine and petrobactin, in addition to decreased production of the siderophore bacillibactin of *B. cereus* in the THOR community [43]. Overall changes in *F. johnsoniae* BGC expression are less pronounced than those in *B. cereus*, but this observation highlights that *P. koreensis* can modulate the SM of its partners via interspecies signals. As a previous study [45] implicated that the antimicrobial koreenceine produced by *P. koreensis* influences the THOR community transcriptome, *P. koreensis* deleted for the koreenceine BGC was tested in the THOR community. Notably, koreenceine inhibits the growth of *F. johnsoniae*. Expression of both petrobactin and bacillibactin BGCs of *B. cereus* is increased in the pairwise and three-member communities containing the *P. koreensis* deleted for the koreenceine BGC compared with the cultures encompassing wild-type *P. koreensis*, while up-regulation of zwittermicin/kanosamine BGC expression is abolished by deletion of the koreenceine BGC in *P. koreensis* [43]. Up-regulation of BGC expression has also been described in another SynCom containing *Burkholderia thailandensis, Chromobacterium subtsugae*, and *Pseudomonas syringae* [46], suggesting a general occurrence of bioactive exometabolite-driven maintenance of competition.

These publications highlight the relevance of simplified microbial communities in dissecting the underlying mechanistic details behind interspecies induction of BGC expression that might emerge in certain combinations of microorganisms. The combination of multi-omics approaches, like those used to investigate the meta-transcriptome and metabolome of THOR community can also be used to predict pathways driving microbe–microbe interactions [47].

## Bioconversion of SM in communities

SynComs are utilized to dissect higher-order interactions, where the function or activity of the community differs from those observed in mono- or pairwise cultures [48]. The emerging function of SynComs can be exploited in environmental applications, e.g., promoting plant growth or protection from pathogens [17,18,41,49]. Simplified microbial communities and laboratory conditions also facilitate the detection of SMs in contrast to highly complex environments that comprise diverse microbial communities.

In addition to specifically detecting the levels of known metabolites of *B. cereus* (zwittermicin/kanosamine, petrobactin, and bacillibactin), metabolomic analysis of the THOR community also revealed molecular features that are strongly associated with the three-member community [43]. Detailed metabolite analysis revealed that the molecular features are connected to the *P. koreensis*-produced cyclic lipopeptide, lokisin. Cyclic lipopeptides consist of a hydrophobic fatty acid covalently linked to a short peptide that is cyclized through a lactone or lactam bond. The cyclic lipopeptide structure creates an amphipathic property that underlies, among others, its membrane-active biological function. An open ring form of lokisin is present in cultures of *P. koreensis* with or without interacting community members of THOR. However, in the presence of *B. cereus*, a deoxy version of the open-ring lokisin is detected [43]. In the three-member community of THOR, cleavage of the terminal aspartic acid and isoleucine moieties from the deoxy-ring-open lokisin is observed, a molecular feature that is absent from *P. koreensis* monoculture or dual-species cultures (*P. koreensis–B. cereus* and *P. koreensis–F. johnsoniae* combinations) [43].

Bioconversion of lipopeptides has also been observed during diverse microbial interactions. As *Bacillus velezensis* and *Streptomyces venezuelae* colocalize on the roots of tomato plants, high proportions of linearized surfactin and iturin are detected [50]. When cultivated on agar-solidified medium, *S. venezuelae* degrades structurally diverse cyclic lipopeptides of the interacting microorganisms, including surfactin, fengycin, and iturin produced by *B. velezensis*, in addition to sessilin, tolaasin, orfamide, xantholisin, and putisolvin of *Pseudomonas* [50]. Based on feature-based molecular networking of mass spectrometry data, degradation of the cyclic lipopeptides is initiated by opening of the peptide cycle, followed by iterative degradation of the linear form and release of free fatty and amino acids. The authors hypothesize that secreted enzymes of *S. venezuelae* are involved in the sequential degradation of cyclic lipopeptides, which is supported by the loss of degradation capacity after heat treatment of *S. venezuelae* cell-free supernatant [50]. Moreover, the degradation products derived from the *Bacillus* lipopeptides support the growth of *S. venezuelae*.

Degradation of lipopeptides could reduce the bioactivity of the SM-producing bacterial strains. *Stenotrophomonas maltophilia* hinders the biocontrol property of *Bacillus* against tomato wilt disease caused by *Ralstonia solanacearum* [51]. Inactivation of tolaasin produced by *Pseudomonas tolaasii*, which causes brown blotch disease of white button mushrooms, has been observed by the fungus-associated helper bacteria of the genus *Mycetocola* (*Mycetocola tolaasinivorans* and *Mycetocola lacteus*) [52]. The description of the metabolic interactions mediated by a lactonase enzyme in this tripartite system highlights how bioconversion functions as an antivirulence strategy. *Rhizoctonia solani* neutralizes *Pseudomonas fuscovaginae* cyclic lipopeptides, syringotoxin and fuscopeptin [53]. *Rhizoctonia solani* secretes two enzymes: an esterase that hydrolyzes the ester bond in syringotoxin and a protease that cleaves the glycine-alanine bond within the peptide backbone of fuscopeptin [53]. *Paenibacillus dendritiformis* degrades surfactin of *B. subtilis*, an SM that *P. dendritiformis* is attracted to, and the degradation products serve as restrictive territorial markers [54]. In other cases, bioconversion of SM might introduce a novel function. *Paenibacillus* degrades syringafactin, a linear lipopeptide of *Pseudomonas*, thereby converting the originally innocuous SMs into compounds that kill amoeba predators [55]. The two dl-peptidases of *Paenibacillus* involved in lipopeptide degradation are induced by the presence of syringafactin and have distinct substrate preferences, while Lip3 acts primarily as a carboxypeptidase, removing a single C-terminal residue, and Lip7 excises a tripeptide [56].

Similarly to the THOR community, a four-member SynCom (Dyrehaven SynCom) has been implicated in a complex bioconversion of the *P. protegens* lipopeptide orfamide A [57]. During establishment of *P. protegens* in the SynCom, the level of orfamide A decreases. Molecular network analysis revealed the hydrolysis of the ester bond connecting the macrocyclic ring in orfamide A, followed by degradation of the linearized product. Pairwise interaction identified *Rhodococcus globerulus* from the SynCom as being responsible for linearization of orfamide A. As orfamide A is important for swarming of *P. protegens*, co-inoculation with *R. globerulus* reduces swarming of the *Pseudomonas* population. Orfamide A subsequent degradation depends on another member of the SynCom, *Stenotrophomonas indicatrix* [57].

Interaction between lipopeptides could directly hinder SM bioactivity. *Bacillus velezensis* mobilizes surfactin not only to promote its surface motility, but this SM also acts as a chemical trap to reduce the toxicity of lipopeptides formed by *Pseudomonas*, like sessilin [58]. A sessilin-producing *Pseudomonas* strain also restricts tomato plant root colonization by *B. velezensis*, excluding the *Bacillus* cells from those parts of the root that are pre-colonized by *Pseudomonas*, unless sessilin production is absent, in which case *Bacillus* co-colonizes the root with *Pseudomonas* [58].

These publications highlight the influence of the endogenous microbial community on the functioning of SM-producing microorganisms, increasing the complexity of the metabolic landscape in nature. The use of SynCom combined with metabolomics could help us to dissect these chemical interactions and their impact on ecosystem functioning.

## Influence of SM on establishment and community composition

Degradation of SMs by interacting species highlights the impact of reduced SM levels on microbial functions, including swarming or plant root colonization. Indeed, SMs equip the producing microorganism with various functions. SMs can be important for bacterial differentiation, like biofilm formation and plant colonization [59–61]; SMs facilitate surface spreading [62–66], in addition to inhibiting other microorganisms [67]. By definition, SMs are metabolites that are not required for the growth of the given producing organisms under simplified laboratory conditions. However, when a bacterium grows in the presence of other microorganisms, SM might be important for the growth of the producing species even if the SM do not have a direct antimicrobial influence on the other microorganisms of the community. In other cases, the antimicrobial or bioactivity effects of SMs determine SynCom assembly [68]. Also, SMs can promote the growth of specific members of microbial communities. A collection of cyclic lipopeptides belonging to the amphisin family produced by *Pseudomonas* sp. SM4 stimulates the growth of the yeast *Candida zeylanoides* in milk, influencing traditional dairy fermentation [69].

Lozano-Andrade and colleagues utilized a simplified soil-mimicking system to cultivate *B. subtilis* and the four members of the Dyrehaven SynCom that allows following SM abundance [70]. In this simplified system, the establishment of *B. subtilis* depends on the production of surfactin [71]. Surfactin has diverse ecological roles [72], including promotion of surface mobility, swarming and sliding, due to its amphiphilic properties [62–66,73], inhibition of plant pathogenic bacteria [74], modulation of biofilm development *in vitro* and during rhizosphere colonization [59–61,75–77], influence on fungal cell morphology and cytoplasmic flow [78–80], and induction of plant immunity [81]. Also, as mentioned above, surfactin acts as a signaling molecule to modulate the production of other SMs [37] and influence the functionality of other SMs [58]. Surfactin delays the growth of closely related genera of *Bacilli* and therefore has specific influence on certain bacterial genera in a soil-derived semi-synthetic community without majorly altering the overall assembly of the community [82]. In the absence of surfactin, the establishment of *B. subtilis* within various SynComs is limited, including the above-mentioned Dyrehaven SynCom and the THOR model community [71]. The Dyrehaven SynCom composition is not modulated by the presence of *B. subtilis*, but surfactin influences the transcriptional landscape and chemodiversity of the community. Specifically, chemical features originating from the SynCom are absent when either surfactin-producing *B. subtilis* strains or purified surfactin are supplemented to the community [71]. The presence of ornithine lipids is observed in the SynCom, which are derived from the outer membrane of Gram-negative bacteria as surrogates of phospholipids under phosphate-limited conditions, while these are reduced in the presence of surfactin. The attenuated establishment of the *B. subtilis* mutant lacking the surfactin BGC is restored by the presence of surfactin-producing strains, suggesting that surfactin is used as a shared good in the population [71]. Indeed, the public good role of surfactin (i.e., it is costly to produce but benefits non-producing strains in a mixed population) has been previously demonstrated during surface spreading and biofilm development [59,65]. As lipopeptides are secreted into the extracellular space and diffuse within the population, they can be shared with non-producing strains [83]. This could explain why bacterial strains can be isolated that lack production of certain lipopeptides [30,84]. Interestingly, *B. subtilis* strains that are unable to produce plipastatin due to the precise deletion of part of the plipastatin BGC have been co-isolated with plipastatin-producing strains from the same gram of soil [84], and the specific plipastatin BGC alteration is conserved in *B. subtilis* isolates obtained on different continents around the world [30]. This suggests that the wide distribution of lipopeptide mutants might be facilitated by the presence of lipopeptide-producing strains in environmental microbiomes. SynComs facilitate the dissection of the role of SMs in complex microbiomes.

## Conclusions

SynComs with defined microbial community composition can be used to reveal the various roles of SMs: how SMs influence the expression of BGCs in the community members, the bioconversion of SMs by the SynCom, and the influence of SMs on establishment in the communities. However, natural environmental microbiomes might also contain SM-producing and non-producing strains, which allows genetic differentiation in their BGCs. Assessing the diverse ecological role of SMs in natural communities requires careful attention that considers the complexity of microbiomes, and it should not be restricted to simple pairwise tests, microbial communities display emerging functions that influence SM lifetime and function. Abiotic factors also influence the functions of the microbiome and the roles of SMs. Finally, the dynamic changes in SM production, including seasonal or daily changes, should also be addressed in the future, as the composition and function of microbiomes are not constant.

## Summary

- SynComs can be used to uncover community-level emerging functions of microbiomes associated with SM production and BGC activation
- Metabolomic analyses of SynComs enable the characterization of SM biotransformation within defined microbial consortia
- SynCom-based approaches facilitate the identification of microbiome-level roles of SM in shaping microbial interactions and community functions.

## Open Access

This article has been published open access under our Subscribe to Open programme, made possible through the support of our subscribing institutions, learn more here: https://portlandpress.com/pages/open_access_options_and_prices#conditional.

## Abbreviations

BGCs: biosynthetic gene clusters
SMs: specialized metabolites
SynComs: synthetic microbial communities
